# Effects of interpretive front-of-pack nutrition labels on food purchases: protocol for the Starlight randomised controlled trial

**DOI:** 10.1186/1471-2458-14-968

**Published:** 2014-09-18

**Authors:** Ekaterina Volkova, Bruce Neal, Mike Rayner, Boyd Swinburn, Helen Eyles, Yannan Jiang, Jo Michie, Cliona Ni Mhurchu

**Affiliations:** National Institute for Health Innovation, University of Auckland, Auckland, New Zealand; The George Institute for Global Health, Sydney, Australia; British Heart Foundation Centre on Population Approaches for Non-Communicable Disease Prevention, Oxford, UK; Epidemiology and Biostatistics, University of Auckland, Auckland, New Zealand

**Keywords:** Nutrition labeling, Mobile applications, Technology, Nutrition policy, Randomized controlled trial (RCT), Traffic-light label, Health star rating label

## Abstract

**Background:**

Interpretive front-of-pack nutrition labels are better understood than non-interpretive labels. However, robust evidence on the effects of such labels on consumer food purchases in the real-world is lacking. Our aim is to assess the effects of two interpretive front-of-pack nutrition labels, compared with a non-interpretive label, on the healthiness of consumer food purchases.

**Methods/Design:**

A five-week (1-week baseline and 4-week intervention) three-arm parallel randomised controlled trial will be conducted using a bespoke smartphone application, which will administer study questionnaires and deliver intervention (Multiple Traffic Light and Health Star Rating) and control (Nutrition Information Panel) labels. To view their allocated nutrition label, participants scan the barcode of packaged food products using their smartphone camera. The assigned label is displayed instantly on the smartphone screen.1500 eligible participants (New Zealand adult smartphone owners who shop in a supermarket at least once a week and are main household shoppers) will be randomised in a 1:1:1 ratio to one of the three nutrition label formats, using computer-generated randomisation sequences. Randomisation will be stratified by ethnicity and interest in healthy eating. Food and beverage purchase data will be collected continuously throughout the study via hard copy till receipts and electronic grocery purchase lists recorded and transmitted using the smartphone application. The primary outcome will be healthiness of food purchases in each trial arm, assessed as mean Food Standards Australia New Zealand nutrient profiling score criterion score for all food and beverages purchased over the intervention period. Secondary outcomes will include saturated fat, sugar, sodium and energy content of food purchases; food expenditure; labelling profile of food purchases (i.e. mean number of Health Star Rating stars and proportion of red, green and amber traffic lights); nutrient profiling score over time and by food categories; purchases of unpackaged foods; self-reported nutrition knowledge and recorded use of assigned labelling system.

**Discussion:**

The Starlight randomised, controlled trial will determine the effects of interpretive front-of-pack nutrition labels on the healthiness of consumer food purchases in the real world.

**Trial registration:**

Australian New Zealand Clinical Trials Registry ACTRN12614000644662 (registered 18 June 2014).

## Background

Obesity and the burden of associated non-communicable disease has been increasing worldwide [1]. Effective, front-of-pack (FOP) nutrition labelling is potentially one of the most cost-effective interventions [2]. However, traditional numerical nutrition labels are difficult to interpret and have limited influence on the average consumer’s food purchasing patterns [3–5].

In New Zealand, the Nutrition Information Panel (NIP), usually found on the back of food packages, is mandatory [6]. A review of nutrition label use found that this is poorly understood by most New Zealanders [7]. Further, use of this nutrition label is particularly low among Māori (indigenous New Zealanders), Pacific, and low-income New Zealanders [8], who experience the highest rates of obesity [9]. Therefore, identifying a labelling format that delivers information effectively to these groups is especially important.

A recent review of New Zealand and Australian food labelling policy recommended introduction of interpretative FOP labels that are easy for consumers to understand and act upon [10]. Substantial global evidence indicates that interpretative labels (using graphics, symbols or colours) are better understood than traditional numeric nutrition labels [11]. However, the impact of such labels on food purchase habits is unclear.

Evaluation of nutrition labelling interventions in the real-world is challenging. Two common approaches are to use controlled settings (for example, a workplace cafeteria or one particular retailer), or consumer surveys. Several cafeteria studies support the ability of FOP labels to promote healthy food choices [12–14]. Surveys and choice experiments also report favourable results, suggesting FOP labels help participants to successfully identify healthier options [15] and are used to make food choices [16–18].

A limited number of studies report on the effect of FOP labels in retail settings. A large observational study conducted by Sacks et al. [19] investigated the effect of supplementary traffic-light FOP labels implemented as a voluntary nutrition labelling system in a UK retailer. The study reported no difference between sales of healthy and unhealthy ready meals and sandwiches following introduction of traffic-light FOP labels, compared to the period prior to label administration. The major limitation of this study was the small sample of products included in the study. Another large intervention study assessed the effectiveness of “Guiding Star” shelf labelling system across a chain of 168 US supermarkets [20]. Analysis of supermarket sales data showed a significant increase in proportion of star-rated product sales and corresponding decrease in sales of un-starred products in same food categories [20]. One limitation however was the lack of a control group within the same stores. Randomised controlled trials are needed to provide robust evidence on the effect of the FOP labels on real-world retail food purchases.

The current study assesses two types of FOP nutrition labels. One is the colour-coded traffic-light (TL) FOP label [21]. This label uses colour-coded categories to reflect low (green), medium (amber) and high (red) content of four nutrients: total fat, saturated fat, total sugar and salt. The underpinning algorithm is that recommended by the UK Governments [22]. This FOP label has been shown to have a high level of understanding and acceptance across major ethnic and income groups [23]. The other label to be evaluated is the new Health Star Rating (HSR) system proposed for implementation in Australia. This label assigns a star rating to a food from ½ (least healthy) to 5 (most healthy) stars based on the underpinning HSR score algorithm [24].

The intervention will be delivered using novel smartphone technology, based on the FoodSwitch free smartphone application (app) where users scan the barcode of a packaged food and receive an immediate, interpretive TL nutrition label on their phone screen, and recommendations for healthier options [25]. A similar smartphone app designed for the current study will be used to deliver TL, HSR or NIP nutrition labels to study participants. The primary aim of the trial is to assess the effectiveness of TL and HSR label formats, compared with the standard NIP, on healthiness of consumer food purchases. The null hypothesis of no difference with the control label will be tested for each of the intervention arms.

## Methods/Design

### Study design

**Figure 1:**
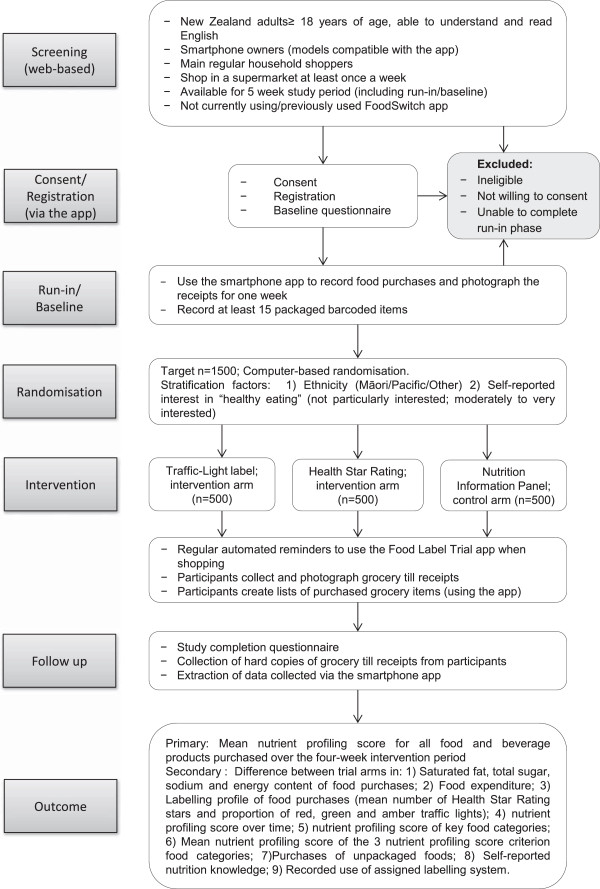
**Flow chart for the Starlight trial.**

Starlight is a three-arm parallel randomised controlled trial (Figure 1). A total of 1,500 participants will be randomised to receive either one of two FOP labels (TL or HSR; intervention arms) or NIP label (control arm) in a 1:1:1 ratio. All nutrition labels will be delivered via a bespoke “Food Label Trial” smartphone app.

### Approval

Ethical approval from the University of Auckland Human Participants Ethics Committee was received on 26 May 2014. The Starlight trial is registered in the Australian New Zealand Clinical Trials Registry (registration number ACTRN12614000644662).

**Intervention arms**

**Figure 2:**
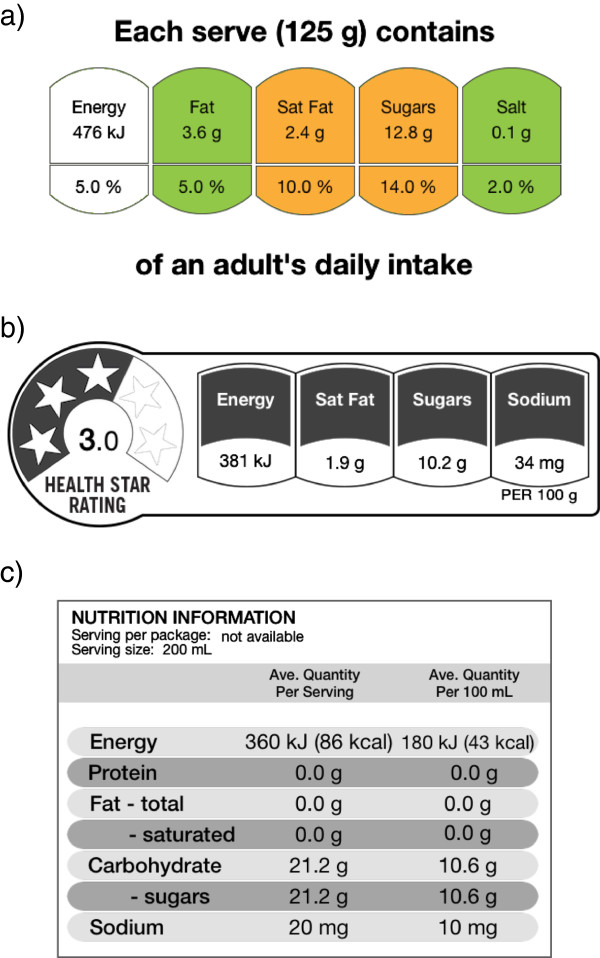
**Example of intervention label formats. a)** Traffic-lights FOP label **b)** Health Star Rating FOP label **c)** New Zealand NIP label.

1. FOP Traffic-Light label (Figure 2a).
2. FOP Health Star Rating label (Figure 2b).

**Control arm:**

1. Standard New Zealand non-interpretive, numerical NIP (Figure 2c)

### “Food Label Trial” smartphone application

All allocated labels will be delivered via the bespoke “Food Label Trial” smartphone app, which enables participants to view the allocated nutrition label for packaged barcoded products. To view the label, users scan barcodes of packaged food products using the smartphone camera, and assigned labels instantly appear on the phone screen. At the same time the app also displays a random selection of other foods in the same food category with same label format to encourage comparative review of available choices and to better test the influence of the label on purchasing decision. If a food item is missing from the app database, participants will receive a default message and will have an option of providing the details of this product (photographs and barcode) in order for it to be added to the database.

Additional app functions enable outcome data collection. Participants will use the app to create electronic lists of purchased food/beverage products and to photograph their grocery till receipts. In order to create the electronic list of purchased items the participants will scan barcodes of the items purchased using their smartphone camera.

### Study population

The Starlight trial will recruit 1500 New Zealand adults (aged 18 years and older) who have a smartphone (iPhone or Android), are main household shoppers (i.e. complete 50% or more of the grocery shopping for their household), and shop at a supermarket at least once a week. Participants must be able to read and understand English, and be available for the full duration of the 5-week trial. Only one person per household can participate in the study. Current or previous FoodSwitch app users will be excluded, because FoodSwitch provides TL labels and recommends healthier food options.

### Recruitment and run-in phase/baseline

Participants will be recruited across New Zealand via advertising in local newspapers and on social media websites, household mail drops, at community venues including supermarkets, and utilising existing research team networks. The aim is to recruit approximately equal numbers of Māori (n = 500), Pacific (n = 500) and other ethnic group participants (n = 500).

Eligible participants will be given access to the “Food Label Trial” app. Informed consent and baseline demographic data will be collected from all study participants via the app.

During the run-in/baseline phase participants will record their food and beverage purchases for one week using the app, and collect and photograph the corresponding till receipts using the smartphone app. At least 15 purchased barcoded grocery items will need to be recorded during this period in order to qualify for study entry. Failure to complete the run-in phase will result in ineligibility.

### Randomisation

Participants who complete the run-in phase successfully will be randomised in a 1:1:1 ratio to one of the three label formats (TL; HSR or NIP), using a central computer-based randomisation system. Blocked randomisation will be used with variable block sizes, stratified by self-identified ethnic group (Māori, Pacific, Other) and self-reported interest in “healthy eating” (not particularly interested; moderately to very interested).

### Blinding

It is not possible to blind trial participants to the intervention. However, participants will only see one type of label for the duration of the trial and will not know what other label formats are being tested in the trial.

### Data collection

The baseline questionnaire will collect demographic details (age, gender, ethnicity, income, education level, family size) and self-reported information on interest in nutrition and healthy eating.

Data on participant food and beverage purchases will be collected throughout the one-week baseline and four-week intervention period. Usage of the labelling function will be automatically recorded by the “Food Label Trial” app. Objective purchase data will be supplied by participants in the following modes: 1) electronic list of scanned purchased items (“Food Label Trial” app function); 2) photographs of corresponding grocery till receipts (“Food Label Trial” app function); 3) hard copies of grocery till receipts (returned by participants at the end of the intervention period). The electronic lists of purchased items will be used as the primary data source of packaged food purchases. The till receipts provide information on price and on purchases of non-barcoded items. Photographs of till receipts will be used as a back-up for missing hard copy till receipts.

All data collected via the “Food Label Trial” will be automatically transmitted via Wi-Fi or 3/4G to the app database, hosted on a remote server, and subsequently extracted by researchers to the study database. Hard copies of till receipts will be mailed by participants to the study centre and the additional data manually entered into the study database.

A follow up questionnaire will collect participant feedback on the app (technical issues, usefulness, self-reported impact on food choices), self-reported compliance with the trial protocol (number of shopping events recorded and till receipts returned, usage of the trial app) and perceived changes in participant’s nutrition knowledge.

Regular reminder messages (3 times per week) will be sent throughout the intervention period to encourage participants to use the app and submit data, and to minimise attrition. At the end of the study participants will be provided with reward vouchers as a compensation for the time and potential costs associated with taking part in the trial.

### Outcomes

The primary outcome of the trial will be the mean nutrient profiling score for all food and beverage products purchased over the four-week intervention period. Nutrient profiling score will be calculated using the Food Standards Australia New Zealand (FSANZ) nutrient profiling standard [26]. Food composition data will be obtained from Nutritrack, a brand-specific processed food composition database that contains comprehensive annually-updated information on New Zealand packaged and fast foods [27]. As a secondary approach, the crude nutrient profiling score will be transformed to a scale of 0–100 consistent across all 3 NPSC category foods. A tertiary approach will also be considered on weighted nutrient profiling score stratified by key food categories.

Secondary outcomes will be the difference between trial arms in:

1. Mean saturated fat, total sugar, sodium and energy content per 100 g food purchases over the four-week intervention period;
2. Mean weekly food expenditure over the four-week intervention period;
3. Labelling profile of food purchases (mean number of HSR stars and proportions of red, green and amber traffic lights) over the four-week intervention period;
4. Mean nutrient profiling score for all food and beverage products purchased each week of the intervention period;
5. Mean nutrient profiling score of key food categories likely to be most impacted by nutrition labelling (e.g. breakfast cereals, cereal bars, pizzas and ready meals);
6. Mean nutrient profiling score of the 3 nutrient profiling score criterion food categories (beverages, fats and oils, all other foods)
7. Mean purchases of unpackaged foods (e.g. fruit and vegetables) in g/100 g;
8. Self-reported nutrition knowledge at follow-up;
9. Use of assigned labelling system as recorded by the Food Label Trial app.

### Sample size

A total sample size of 1,500 participants (n = 500 per arm) will have at least 80% power (alpha = 0.05) to detect a minimum 2-unit difference in the mean nutrient profiling score between either of the intervention arms and control with adjustment for multiple comparisons. A 2-unit change in nutrient profiling score is approximately equivalent to the following changes in nutrient content per 100 g food: 78 kJ energy, 0.95 g saturated fat, 1.5 g total sugars and 73 mg sodium (unpublished data). The nutrient profile score will be estimated using the FSANZ nutrient profiling scoring calculator, where food scores span a range of -17 to 53 (a lower score is healthier) [28]. The power estimate assumes a standard deviation of 9.9 based on distribution of >25,000 foods in an Australian food database.

### Statistical analyses

Statistical analyses will be performed using SAS version 9.3 (SAS Institute Inc. Cary NC). All statistical tests will be two-tailed and maintained at a 5% significance level. The baseline characteristics of all study participants will be summarised and tabulated using means (standard deviations, medians and ranges) and frequencies (proportions). Analysis of covariance (ANCOVA) regression models will be used to compare mean nutrient profiling score between intervention and control groups, adjusting for baseline nutrient profiling score and stratification factors. A similar approach will be used for continuous secondary outcomes. Generalized linear models will be used for secondary categorical outcomes. No imputation will be undertaken. Repeated measures mixed models will be used to evaluate treatment effects over time. Sub-group analyses will test possible interactions of the labelling intervention with key food categories, ethnicity (Maori, Pacific, Other), income tertile, and baseline self-reported interest in “healthy eating”. Sensitivity analyses will be undertaken using data only from participants who return at least 75% of till receipts/food purchase data based on pre-randomisation usual reported number of shopping episodes. A statistical analysis plan will be prepared by the trial statistician prior to the final data lock. Reporting will adhere to the CONSORT 2010 guidelines for reporting parallel group randomised trials.

## Discussion

The aim of the Starlight RCT is to measure the effects of two interpretive FOP nutrition labels, compared with the standard NIP, on the healthiness of food purchases. To our knowledge, this is the first RCT assessing the impact of interpretive FOP labels on objectively measured consumer purchases in real-world retail outlets nationwide, without restriction to a particular store or setting. The unique smartphone app designed for the trial will allow shoppers to view nutrition labels of barcoded food products in any retail outlet. The randomised controlled design of the Starlight trial enables use of the NIP label at an individual level as a control, rather than using a control store. The advantage of this approach is that it minimises confounding effects of patterns of sales in different retailers. Another advantage is that it neutralises any effect of using the smartphone app to scan products. The “Food Label Trial” smartphone app will also allow objective assessment of nutrition label use when shopping since this information will be collected automatically by the app. The Starlight trial will also assess the impact, utility and acceptability of proposed label format for Māori and Pacific adults. This is of particular importance, considering the high prevalence of obesity and nutrition-related disease among those groups [9]. According to study by Signal et al. [8], self-reported use of nutrition labels is low among those groups, and both claim to favour simpler nutrition labels that are easier to understand. Whilst FOP labels are the focus of much government, industry and advocacy group attention worldwide, their impact on consumers’ behaviour is uncertain. This large, randomised, controlled trial will provide robust evidence of the effectiveness and potential cost-effectiveness of FOP labelling as means to improve population diets and health.

### Trial status

Recruiting.

## Abbreviations

FOP: Front-of-pack
NIP: Nutrition information panel
App: Application
TL: Traffic-light
HSR: Health star rating
RCT: Randomised controlled trial.

## References

[1] **World Health Organisation** http://www.who.int/gho/ncd/risk_factors/overweight_text/en/index.html

[2] Gortmaker SL, Swinburn BA, Levy D, Carter R, Mabry PL, Finegood DT, Huang T, Marsh T, Moodie ML (2011). Changing the future of obesity: science, policy, and action. Lancet.

[3] Temple NJ, Fraser J (2013). Food labels: a critical assessment. Nutrition.

[4] Cowburn G, Stockley L (2005). Consumer understanding and use of nutrition labelling: a systematic review. Public Health Nutr.

[5] Grunert KG, Fernández-Celemín L, Wills JM, Storcksdieck Genannt Bonsmann S, Nureeva L (2010). Use and understanding of nutrition information on food labels in six European countries. Z Gesundh Wiss.

[6] Australia New Zealand Food Standards Code - Standard 1.2.8 (2013). Nutrition information requirements.

[7] Ni Mhurchu C, Gorton D (2007). Nutrition labels and claims in New Zealand and Australia: a review of use and understanding. Aust N Z J Public Health.

[8] Signal L, Lanumata T, Robinson JA, Tavila A, Wilton J, Ni Mhurchu C (2008). Perceptions of New Zealand nutrition labels by Māori, Pacific and low-income shoppers. Public Health Nutr.

[9] Ministry of Health (2013). New Zealand Health Survey: Annual Update of key Findings 2012/13.

[10] Blewett N, Goddard N, Pettigrew S, Reynolds C, Yeatman H (2011). Labelling Logic Review of Food Labelling law and Policy.

[11] Campos S, Doxey J, Hammond D (2011). Nutrition labels on pre-packaged foods: a systematic review. Public Health Nutr.

[12] Vyth EL, Steenhuis IHM, Heymans MW, Roodenburg AJC, Brug J, Seidell JC (2011). Influence of placement of a nutrition logo on cafeteria menu items on lunchtime food choices at Dutch work sites. J Am Diet Assoc.

[13] Thorndike AN, Sonnenberg L, Riis J, Barraclough S, Levy DE (2012). A 2-phase labeling and choice architecture intervention to improve healthy food and beverage choices. Am J Public Health.

[14] Levy DE, Riis J, Sonnenberg LM, Barraclough SJ, Thorndike AN (2012). Food choices of minority and low-income employees: a cafeteria intervention. Am J Prev Med.

[15] Watson WL, Kelly B, Hectord D, Hughesa C, Kingd L, Crawforde J, Sergeante J, Chapmana K (2014). Can front-of-pack labelling schemes guide healthier food choices? Australian shoppers’ responses to seven labelling formats. Appetite.

[16] Balcombe K, Fraser I, Di Falco S (2010). Traffic lights and food choice. A choice experiment examining the relationship between nutritional food labels and price. Food Policy.

[17] Hieke S, Wilczynski P (2012). Colour Me In - an empirical study on consumer responses to the traffic light signposting system in nutrition labelling. Public Health Nutr.

[18] Steenhuis I, van Assema P, Reubsaet A, Kok G (2004). Process evaluation of two environmental nutrition programmes and an educational nutrition programme conducted at supermarkets and worksite cafeterias in the Netherlands. J Hum Nutr Diet.

[19] Sacks G, Rayner M, Swinburn B (2009). Impact of front-of-pack ‘traffic-light’ nutrition labelling on consumer food purchases in the UK. Health Promot Int.

[20] Sutherland LA, Kaley LA, Fischer L (2010). Guiding stars: the effect of a nutrition navigation program on consumer purchases at the supermarket. Am J Clin Nutr.

[21] Food Standards Agency (2007). Front of Pack Nutritional Signpost Labelling: Technical Guidance.

[22] **Guide to Creating a Front of Pack (FoP) Nutrition Label for pre-Packed Products Sold Through Retail Outlets** In *The Department of Health (UK), the Food Standards Agency (UK), and devolved administrations in Scotland, Northern Ireland and Wales in collaboration with the British Retail Consortium*. UK: Department of Health; 2013.

[23] Gorton D, Ni Mhurchu C, Chen MH, Dixon R (2009). Nutrition labels: a survey of use, understanding and preferences among ethnically diverse shoppers in New Zealand. Public Health Nutr.

[24] **Health Star Rating – new food labelling system** http://www.foodsafety.govt.nz/industry/general/labelling-composition/health-star-rating/

[25] **FoodSwitch** http://www.foodswitch.co.nz/

[26] **Food Standards Australia New Zealand** http://www.comlaw.gov.au/Details/F2013L00054

[27] Rosentreter SC, Eyles H, Ni Mhurchu C (2013). Traffic lights and health claims: a comparative analysis of the nutrient profile of packaged foods available for sale in New Zealand supermarkets. Aust N Z J Public Health.

[28] **Food Standards Australia New Zealand** http://www.foodstandards.gov.au/consumerinformation/nutritionhealthandrelatedclaims/nutrientprofilingcal3499.cfm

[CR29] The pre-publication history for this paper can be accessed here:http://www.biomedcentral.com/1471-2458/14/968/prepub

